# The psychological well-being of family caregivers of autistic people during the COVID-19 lockdown in Italy

**DOI:** 10.1192/j.eurpsy.2022.526

**Published:** 2022-09-01

**Authors:** M. Martinez, L. Fusar-Poli, V. Meo, F. Patania, T. Surace, C. Avanzato, E. Aguglia, M.S. Signorelli

**Affiliations:** University of Catania, Department Of Clinical And Experimental Medicine, Catania, Italy

**Keywords:** Covid-19, psychological distress, caregivers, autism spectum disorder

## Abstract

**Introduction:**

People with autism spectrum disorder (ASD) frequently need support due to the elevated prevalence of psychiatric and medical comorbidities. The Covid-19 outbreak has severely affected the routinary functioning of healthcare services, thus causing severe consequences for autistic people and their caregivers, an already fragile population prone to mental health diseases.

**Objectives:**

1. To compare the levels of psychological well-being, insomnia, and family distress perceived by caregivers of autistic people to those perceived by caregivers of people with other types of disability. 2. To evaluate predictors of individual and family distress reported by caregivers of autistic individuals.

**Methods:**

We collected data through a cross-sectional web-based observational study from April 19 to May 3, 2020. Socio-demographic information were collected, and psychopathological variables were assessed using the General Health Questionnaire-12, the Insomnia Severity Index, the Brief Resilient Coping Scale, and the Family Distress Index.

**Results:**

No significant differences emerged between the two groups of caregivers in terms of well-being, sleep quality, family distress, and level of resilience. The risk of individual distress during the pandemic was higher in people caring for younger autistic people. Lower levels of resilience predicted higher levels of individual distress among caregivers of autistic people.

**Conclusions:**

Our study confirmed that caregivers’ mental health is worthy of attention and that people with disabilities may benefit for well-organized healthcare support networks (e.g. in-home services). The non-significant differences found between caregivers of ASD and non-ASD individuals may be related to the severe distress that Covid pandemic caused on the entire population.

**Disclosure:**

No significant relationships.

